# Digital Health Intervention Effect on Older Adults With Chronic Diseases Living Alone: Systematic Review and Meta-Analysis of Randomized Controlled Trials

**DOI:** 10.2196/63168

**Published:** 2025-03-31

**Authors:** Yoonseo Park, Eun-Ji Kim, Sewon Park, Munjae Lee

**Affiliations:** 1 Department of Convergence Healthcare Medicine Ajou University Suwon-si, Gyeonggi-do Republic of Korea; 2 Department of Medical Science School of Medicine Ajou University Suwon-si, Gyeonggi-do Republic of Korea

**Keywords:** digital health, chronic diseases, older adults, independent living, aging, chronic disease, living alone, self-management, medical cost, cost management, health promotion, effectiveness, quality of life, physical activity, health care, health informatics, systematic review, meta-analysis, PRISMA

## Abstract

**Background:**

The incidence of chronic diseases is increasing owing to the aging population; in particular, older adults living alone struggle with self-management and medical expenses. Digital health can contribute to medical cost management and health promotion, but its effectiveness for older adults living alone remains unclear. In a rapidly aging society, it is important to demonstrate the effect of digital health on improving the lives of older adults living alone and reducing the burden of chronic diseases.

**Objective:**

This study aims to examine the intervention effects of digital health on self-management, quality of life, and medical factors for older adults living alone with common chronic diseases such as cardiovascular disease, respiratory disease, and musculoskeletal disorders through a systematic literature review and meta-analysis.

**Methods:**

We searched the literature using 3 databases, including PubMed, CINAHL, and Cochrane CENTRAL, for literature published in overseas academic journals up to October 2024. The final 11 papers were used for analysis based on selection and exclusion criteria. Meta-analysis was used to calculate the mean difference and standardized mean difference (SMD) for the selected literature using RevMan (version 5.4; Cochrane). The effect size and heterogeneity were calculated through 95% CI.

**Results:**

As a result of conducting a meta-analysis of 8 of 11 documents, there was a significant effect of self-management factors on moderate-to-vigorous physical activity (SMD=0.08; *z*=2.07; *P*=.04). However, among self-management factors, low-density lipoprotein cholesterol (SMD=–0.04; *z*=0.91; *P*=.36) did not show statistically significant results. Among the medical factors, general quality of life (SMD=0.11; *z*=0.93; *P*=.35), depression (SMD=–3.95; *z*=1.59; *P*=.11), and hospital days (SMD=–1.57; *z*=0.91; *P*=.36) also did not show statistically significant results. However, it was confirmed that they improved after a digital health intervention.

**Conclusions:**

This study demonstrated that digital health interventions are effective in improving physical activity in older adults with chronic diseases living alone. However, owing to the characteristics of older adults living alone, there is a need to further expand digital health to combine care services that can manage diseases at home.

## Introduction

According to the United Nations, older adults aged 65 years and older are expected to account for 16% of the world’s population in 2050, when the risk of interpersonal, cognitive, and physical decline caused by aging will gradually become more prominent [1,2]. Owing to aging, the number of older adults living alone is continuously increasing, and various social problems require satisfying basic human needs, such as health care and others [3,4]. Aging, disease, and an increase in chronic diseases are natural phenomena, but the health of older adults living alone must be considered from multifaceted and physical perspectives [5,6]. Functional status refers to activities of daily living and the ability to adapt to a given environment; older adults living alone can be exposed to more health risk factors and have a lower functional status than older adults living together [7,8]. Furthermore, because older adults living alone tend to perceive their own subjective health status as lower than that of older adults living together, improving the health of older adults living alone is emerging as an important task [9]. In particular, the chronic diseases and mental health conditions of older adults living alone, such as feelings of alienation, loneliness, and a sense of isolation, are likely to worsen due to the isolation problem following the COVID-19 pandemic; it is necessary to establish a medical system that can manage the daily lives of older adults living alone [10,11].

For this reason, measures to manage and treat diseases using digital health are being actively implemented. Digital health refers to personalized health care and health management services that combine health care with information and communication technology. It is used to manage medical conditions virtually through telemedicine, wearable devices, and mobile health (mHealth) apps [12,13]. The introduction of digital health services can reduce medical costs by improving the efficiency of medical services [14]. Telemedicine and online consultations enable smooth communication with medical experts, reducing medical and travel costs and ultimately alleviating the burden of medical expenses. In this way, digital health enables older adults to manage their health comfortably from home, alleviating the physical burden of accessing health care services and ultimately improving health-related quality of life [15,16]. In the case of older adults, digital literacy is needed to maximize the effectiveness of digital health, which refers to the ability to understand and use digital tools and technologies [17]. Through this capability, older people can check their health status in real time and monitor and manage their personal health data. This can enhance their self-management abilities, contribute to preventive health care, and support the continuous management of chronic disease [18,19].

For adults with type 2 diabetes mellitus, Aminuddin et al [20] examined the effectiveness of a smartphone app on self-efficacy, self-management activities, health-related quality of life, glycated hemoglobin, BMI, and blood pressure levels, finding that it led to improvements in health behaviors and enhanced the effectiveness of self-management activities [20]. Furthermore, Ma et al [21] conducted a systematic literature review and meta-analysis of the effectiveness of telemedicine in patients with chronic diseases. They found that the telemedicine intervention improved systolic blood pressure in hypertensive patients and glycated hemoglobin and fasting blood sugar in diabetic patients and had a positive effect on improving negative sentiments and managing medication compliance in patients with rheumatoid arthritis [21]. Although studies have been conducted confirming the effectiveness of digital health in chronic disease management, there is a lack of research verifying the effectiveness of digital health interventions for older adults with chronic diseases living alone.

Therefore, there is a need to prepare grounds to verify the effectiveness of digital health for the healthy lives of older adults living alone and those with chronic diseases, which will increase in the future, and to increase the use of digital health. This study aims to examine the effects of digital health interventions on self-management, quality of life, and medical factors in older adults living alone with prevalent chronic conditions such as cardiovascular, respiratory, and musculoskeletal diseases through a systematic literature review and meta-analysis. Through these findings, this study seeks to contribute to the development of strategies for using digital health to predict and manage the health conditions of older adults living alone.

## Methods

### Research Design

This study is a systematic literature review and meta-analysis that integrates research results on the effectiveness of digital health interventions for older adults with chronic diseases living alone. To use the search strategy, the study was conducted based on the systematic literature review manual approach of the National Evidence-based Healthcare Collaborating Agency and the guidelines and flow charts of PRISMA (Preferred Reporting Items for Systematic Reviews and Meta-Analyses; see PRISMA checklist in Multimedia Appendix 1). Published data were used in this study and in the preparation of this paper; hence, no ethics approval was required.

### Eligibility Criteria

The specific criteria used to conduct a systematic literature review were as follows: (1) the populations were older adults with chronic diseases living alone, (2) the intervention referred to all digitized health interventions, (3) the comparison focused on older adults living alone who are provided with general and active management, and (4) the outcomes contained self-management factors including blood pressure, cholesterol, and physical activity management; quality of life factors including depression and general quality of life; and medical factors including hospital days. This study included randomized controlled trials and excluded cases and protocols.

### Data Search and Selection Process

The literature collection was not limited by publication year; studies published up to October 2024 were searched. The literature was collected using a total of 3 web search engines: PubMed, the Cumulative Index to Nursing and Allied Health Literature, and Cochrane CENTRAL, which are databases that can search for studies in the fields of medicine, nursing, and health care systems. Since we used reliable major databases, we did not perform separate manual searches, but we performed a systematic search strategy using thorough search formulas. The keywords used to search the literature were (Chronic Disease OR Comorbidity OR Chronic Condition) AND (Aged OR Aging) AND (Community OR Independent Living) AND (Digital Health OR Digital Medicine OR Telehealth OR Smart Health). The selection criteria were as follows: (1) studies on the effectiveness of digital health interventions for older adults with chronic diseases living alone, (2) experimental studies on randomized control groups, and (3) academic papers published in academic journals. The exclusion criteria were as follows: (1) studies not written in English, (2) inappropriate experimental research designs, such as case studies or literature reviews, and (3) participants aged 65 years and younger.

### Quality and Evidence Assessment

After the selection process, Cochrane’s Risk of Bias Tool was used to evaluate the quality of the selected studies, which was conducted independently by 2 researchers [22]. The assessment consisted of 7 evaluation areas; each question was evaluated by dividing the risk of bias as low, clear, or high based on the information described in the literature. Disagreements were resolved through discussions. The risks were assessed to create a random allocation sequence, concealment of the allocation sequence, blinding of participants and researchers, blinding of outcome evaluators, insufficient data, selective reporting of results, and other potentialities that threatened validity.

### Data Analysis

After analyzing the properties of the literature included in the systematic literature review, 2 researchers (YP and EJK) organized the data through discussion according to the data extraction form. The data extraction form included general properties of the literature: author, year of publication, country, research design, research subjects, intervention methods, outcome variables (measurement tools), and research results. In cases where a 95% CI was reported instead of the SD, the CI was calculated using the formula in the Cochrane Handbook of Systematic Reviews [23].

In this study, the Cochrane Library’s RevMan (version 5.4) program was used to conduct the meta-analysis, and heterogeneity tests and effect sizes were calculated and presented. Since the selected literature was judged to have heterogeneity between the diagnosis of subjects, type of digital health intervention, and measurement tools for outcome variables, it was difficult to assume the effect size of a single treatment; thus, they were analyzed using a random-effect model. Furthermore, for the measurement scale of the studies, the mean difference and standardized mean difference (SMD) were used depending on whether the same or different scales were adopted, in which the 95% CI was used. The inverse of variance was used as the weight of each effect size [24].

To evaluate the statistical heterogeneity of the effect size of the selected literature, the chi-square test was performed after calculating the Q value (ie, the total observed variance); the measurement was conducted using the *P* value and *I*². *I*² is the index representing the variance ratio between actual studies to total variance, in which if the *P* value is less than 0.10 and *I*² is 50% or higher, the heterogeneity of the effect size is considered significant [25].

## Results

### Identification of Studies

**Figure 1:**
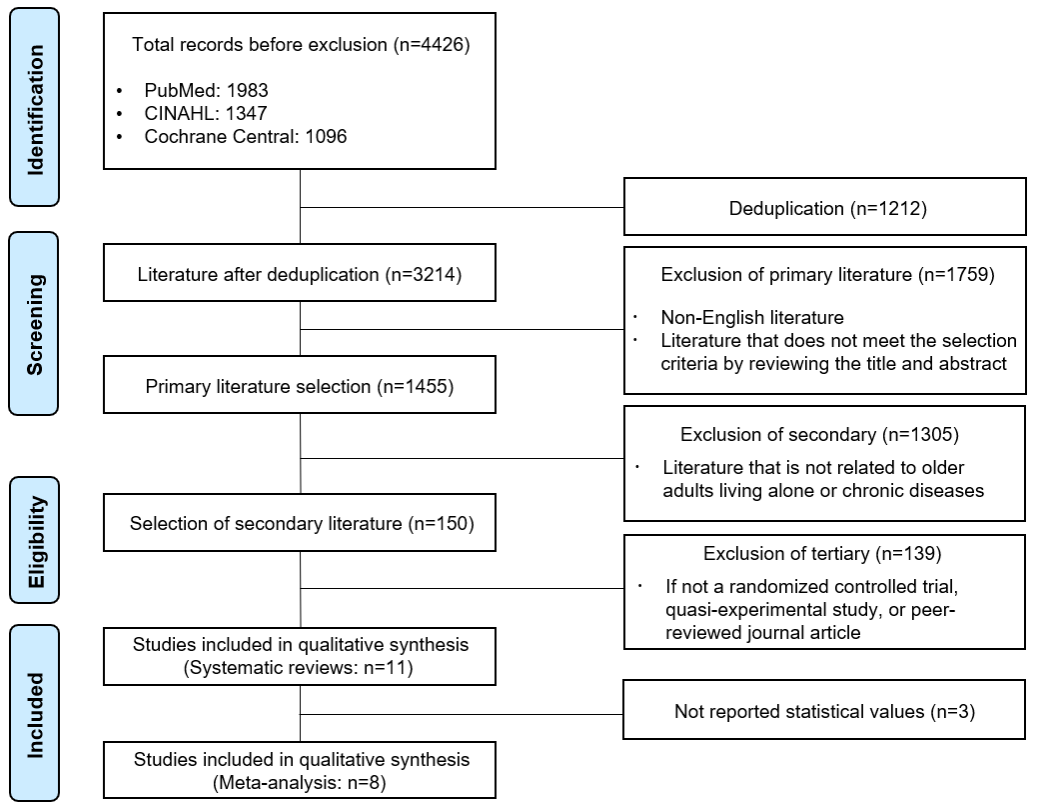
PRISMA (Preferred Reporting Items for Systematic Reviews and Meta-Analyses) flow diagram of study selection and screening process.

A total of 2 researchers independently searched the literature in overseas databases and retrieved 4426 articles, of which 1212 duplicate documents were deleted using the Endnote 20 program (Clarivate) or manually. Subsequently, the titles and abstracts of the papers were reviewed to select and exclude primary literature. The full texts of the literature selected in the primary selection process were secured, and the original text was reviewed by applying the previously defined selection and exclusion criteria for the literature to select the secondary literature. The literature included in the final assessment was selected through agreement between the 2 researchers; in cases where the 2 researchers could not reach an agreement, they reevaluated the literature after sufficient discussion and then reached a consensus. Subsequently, 11 papers were selected through a quality assessment of the literature for systematic literature reviews. Among them, 8 papers were included in the meta-analysis after excluding 3 articles that did not present the statistical values required for meta-analysis (Figure 1).

### Characteristics of Included Studies

Regarding the general characteristics of the literature, they are randomized controlled trial studies; 9 papers were found that were conducted in a single country (1 each in France, Canada, the Netherlands, Italy, China, and Hong Kong, and 3 in the United States) [26-34], while 2 were multinational studies (1 in the Netherlands, Finland, and France, and 1 in Spain, the United Kingdom, Slovenia, Estonia, and Sweden) [35,36]. The mean age of participants was 72 (SD 5.71) years. All participants were older adults with chronic diseases living alone. The most frequent chronic diseases were hypertension, diabetes, and cardiovascular disease.

Among the 11 papers, 6 were on self-management factors [26,28,30,31,34,36], 2 were on quality of life factors [33,35], and 3 were on medical factors [27,29,32], of which self-management factors were the most important. The types of digital health interventions for self-management factors were divided into remote monitoring, interactive internet intervention, home telehealth, mobile health, and eHealth. The main outcome variables were blood pressure, low-density lipoprotein (LDL) cholesterol, 24-hour ambulatory blood pressure monitoring, depression or anxiety symptoms, health status, physical activity, lifestyle habits, and falling accident incidence rate.

In addition, the type of digital health intervention in medical factors was remote monitoring, and the main outcome variables included rehospitalization period and cumulative incidence, health and functional status, patient satisfaction, and health care use data. The type of digital health intervention in quality of life factors was remote monitoring, in which the main outcome variables were a chronic obstructive pulmonary disease assessment test, depression status (Patient Health Questionnaire-9), and a quality of life (EuroQol-5 Dimension) survey. The results of the data analysis of the 11 papers included in this study are presented in Table 1.

**Table 1:** Characteristics of the studies.

Author (year)	Country	Study design	Participant characteristics	Disease	Interventions	Main outcome variables (measurement tools)
Tchalla et al [27] (2023)	France	RCT^a^	Average age 80.3 years; intervention group of 237 personnel; control group of 237 personnel	COPD^b^, diabetes, hypertension, chronic kidney failure, stroke, neurodegenerative disease	Remote monitoring	Cumulative incidence of readmission Clinical outcomes of readmission
Lau et al [30] (2022)	Canada	RCT	Average age 79.5 years; intervention group of 47 personnel; control group of 45 personnel	Hypertension	Remote monitoring	24 hours a day, ABPM^c^, and HBPM^d^ Quality of life: EuroQol-5 Dimension Depression or anxiety: PHQ-8^e^ and GAD-2^f^
Kwan et al [31] (2020)	Hong Kong	RCT	Average age 71 years; intervention group of 16 personnel; control group of 17 personnel	Cognitive impairment and dementia progression stages	Mobile health	Physical activity: Montreal cognitive assessment, Fried frailty index, Physical activity Scale for the elderly, 6-minute walk test, and moderate-to-vigorous physical activity
Volders et al [26] (2020)	The Netherlands	RCT	Average age 74.5 years; intervention group of 260 personnel; control group of 325 personnel	Musculoskeletal and back disorder, COPD, rheumatism, osteoporosis, chronic heart disease	eHealth	Self-Management: Short questionnaire to assess health-enhancing physical activity Physical activity: physical activity, leisure-time physical activity, and moderate-to-vigorous physical activity
Richard et al [36] (2019)	The Netherlands, Finland, and France	RCT	Average age 69 years; intervention group of 1389 personnel; control group of 1335 personnel	Cardiovascular risk factors, cardiovascular disease, and diabetes	Interactive internet intervention	Health status: systolic blood pressure, LDL^g^ cholesterol, BMI^h^, glycated hemoglobin A1c, and physical activity (hours per week) Lifestyle habits: dietary intake and smoking cessation
Bernocchi et al [34] (2019)	Italy	RCT	Average age 79 years; intervention group of 100 personnel; control group of 100 personnel	One or more chronic conditions (cardiac, respiratory, neuromuscular, or neurological)	Home-telehealth	Fall incidence rate Change in functional status: T1-T0 Daily life: Activities of daily living, Barthel index, and instrumental activities of daily living scale
Sun et al [28] (2019)	China	RCT	Average age 68 years; intervention group of 44 personnel; control group of 47 personnel	Type 2 diabetes	Mobile health	Physical activity information: provided by the patient (training on how to send pedometer data to staff by text message) Health information: FBG^i^, PBG^j^, glycated hemoglobin A1c, TC^k^, TG^l^, HDL-C^m^, LDL-C, BMI, and blood pressure (systolic and diastolic)
Walker et al [35] (2018)	Spain, England, Slovenia, Estonia, and Sweden	RCT	Average age 71 years; intervention group of 154 personnel; control group of 158 personnel	COPD	Remote monitoring	COPD assessment: COPD Assessment Tool Score Patient Health Questionnaire-9 score Quality of life: EuroQol-5 Dimension
Gellis et al [32] (2014)	United States	RCT	Average age 79 years; intervention group of 57 personnel; control group of 58 personnel	Chronic disease (congestive heart failure, chronic obstructive pulmonary disease)	Remote monitoring	Depression status: Hamilton depression rating scale, Patient Health Questionnaire-9 Health and functional status: 12-item short-form survey Problem-solving coping skills: social problem-solving inventory—revised Patient satisfaction: satisfaction survey Health care use data: electronic medical record
Gellis et al [33] (2012)	United States	RCT	Average age 79 years; intervention group of 51 personnel; control group of 51 personnel	Heart failure, COPD	Telehealth	Depression status: Center for Epidemiologic Studies Depression and Patient health questionnaire Quality of life: Short form survey of (1) general health, (2) body pain, and (3) social function Patient satisfaction survey: satisfaction with the telehealth service
Soran et al [29] (2010)	United States	RCT	Average age 76.39 years; intervention group of 160 personnel; control group of 155 personnel	Heart failure	Remote monitoring	Cost of medical services: Medicare claims data information

^a^RCT: randomized controlled trial.

^b^COPD: chronic obstructive pulmonary disease.

^c^ABPM: ambulatory blood pressure monitoring.

^d^HBPM: home blood pressure monitoring.

^e^PHQ-8: Patient Health Questionnaire-8.

^f^GAD-2: Generalized Anxiety Disorder 2.

^g^LDL: low-density lipoprotein.

^h^BMI: body mass index.

^i^FBG: fasting blood glucose.

^j^PBG: postprandial blood glucose.

^k^TC: total cholesterol.

^l^TG: triglyceride.

^m^HDL-C: high-density lipoprotein—cholesterol.

### Quality Assessment

**Figure 2:**
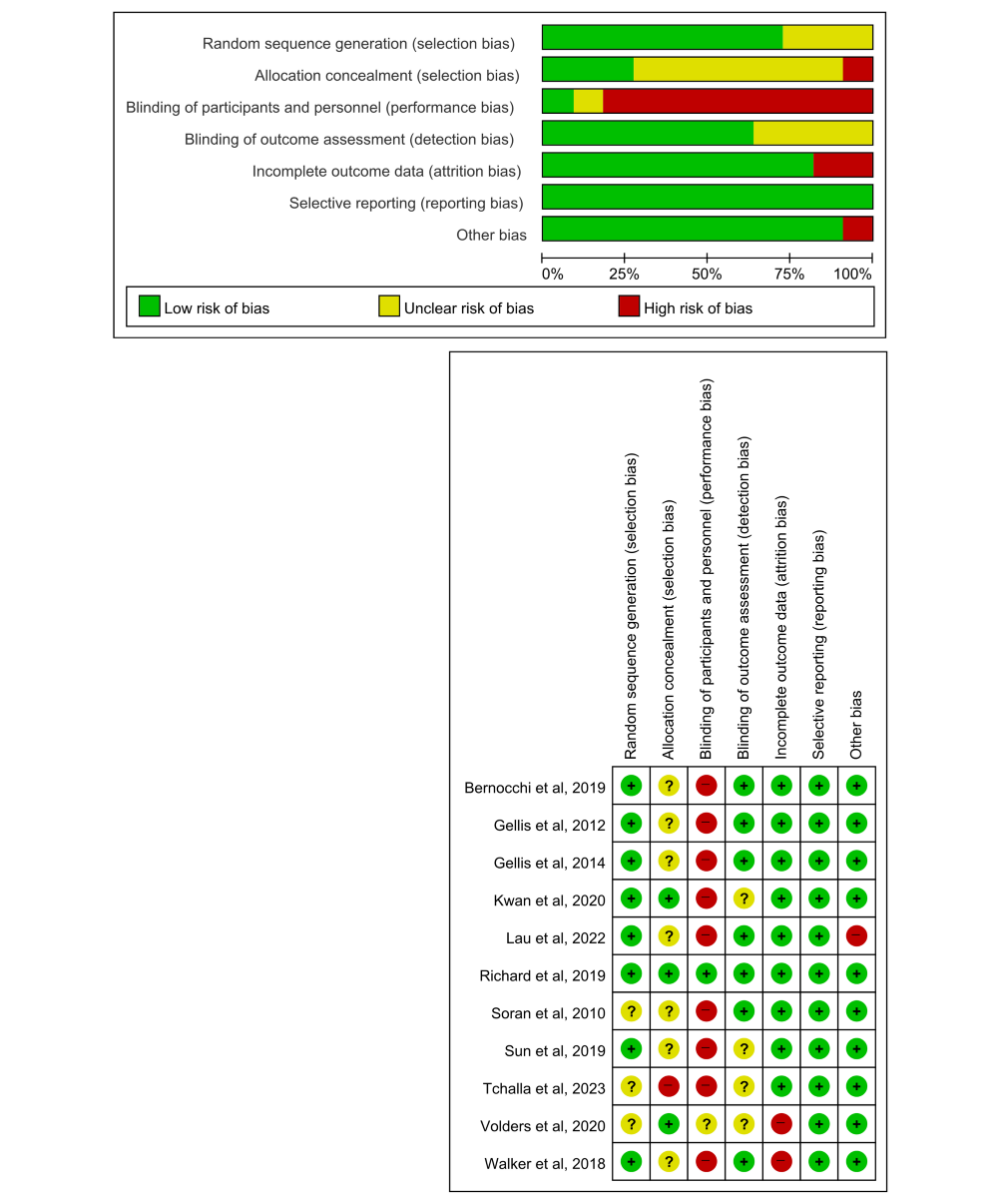
Risk of bias summary and graph [34,33,32,31,30,36,29,28,27,26,35].

In this study, we assessed the quality of the literature based on random sequence generation, allocation concealment, blinding of participants and personnel, blinding of outcome assessment, incomplete outcome data, selective reporting, and other biases. For the 11 selected papers, we presented a summary and graphs of the assessment results (Figure 2). Among the selected papers, 10 had at least one component with a high risk of bias (90.9%). In particular, due to the properties of the application of the intervention method, the blinding of study participants and personnel was impossible, which may have influenced the behavioral results of the research participants and results; thus, the risk of bias was assessed as high at 81.8%.

Furthermore, because 8 papers did not perform allocation concealment or provided insufficient information to determine the risk of bias, the risk of bias for the relevant items was evaluated as high or unclear (72.7%). In other areas, the risk of bias for the literature was low (random sequence generation: 72.7%, blinding of outcome assessment: 63.6%, incomplete outcome data: 81.8%, selective reporting: 100%, and other: 90.9%).

### Digital Health Care Intervention Effect

In this study, we conducted a meta-analysis of the effectiveness of digital health interventions for older adults with chronic diseases living alone. Among the 11 papers selected, the effects were assessed on self-management, quality of life, and medical factors for 8 papers that presented statistical values necessary for meta-analysis, for which the measurement results were visualized using a forest plot. Furthermore, a subanalysis was conducted by dividing the detailed factors of the measured results.

### Self-Management Factor Effects of Digital Health Care Intervention

The effectiveness of digital health interventions on self-management was assessed in 4 papers [26,28,31,36]. In this subgroup, systolic blood pressure, LDL cholesterol level, and moderate-to-vigorous physical activity were divided and then evaluated.

#### Systolic Blood Pressure

**Figure 3:**
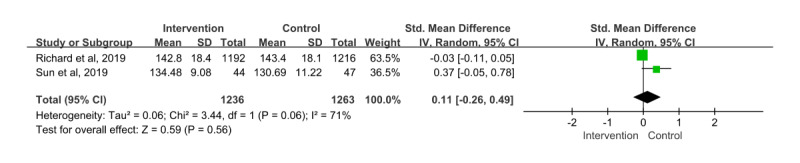
Effect size of systolic blood pressure (SBP). IV: inverse variance [36,28].

A total of 3 papers [28,30,36] were measured on systolic blood pressure. Among them, the paper by Lau [30] was excluded because it measured the target achievement rate of systolic blood pressure for 24-hour ambulatory blood pressure monitoring. Therefore, the remaining 2 papers were included in the meta-analysis; a total of 2499 patients participated and provided information on systolic blood pressure. The SMD for this showed an effect size of 0.11 (95% CI –0.26 to 0.49). In addition, the effects of the intervention and control groups were not statistically significant (*z*=0.59; *P*=.56); the heterogeneity among the articles was considerable (*χ*^2^_1_=3.44; *P*=.06; *I*²=71%; Figure 3).

#### LDL Cholesterol

**Figure 4:**
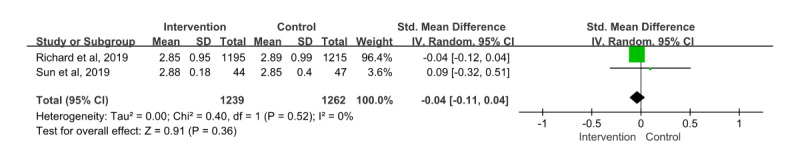
Effect size of low-density lipoprotein (LDL)-cholesterol. IV: inverse variance [36,28].

A total of 2 papers [28,36] were included in the meta-analysis, in which 2501 patients participated and provided information on LDL cholesterol. The SMD for this showed an effect size of –0.04 (95% CI –0.11 to 0.04). Furthermore, the effects of the intervention and control groups were not statistically significant (*z*=0.91; *P*=.36), and heterogeneity among the studies was low (*χ*^2^_1_=0.40; *P*=.52; *I*²=0%; Figure 4).

#### Moderate-to-Vigorous Physical Activity

**Figure 5:**
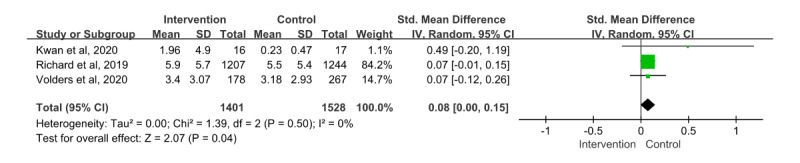
Effect Size of moderate-to-vigorous physical activity (MVPA). IV: inverse variance [31,36,26].

In total, 3 papers [26,31,36] were included in the meta-analysis; 2929 patients participated and provided information on moderate-to-vigorous physical activity. The SMD showed an effect size of 0.08 (95% CI 0.00-0.15). Furthermore, the effect of the intervention and control groups was statistically significant (*z*=2.07; *P*=.04), and the heterogeneity among the papers was low (*χ*^2^_2_=1.39; *P*=.50; *I*²=0%; Figure 5).

### Impact of Digital Health Care Intervention on Quality of Life Factors

#### Overview

In 4 papers [32-35], the effectiveness of digital health interventions was evaluated based on quality of life factors. As subgroups, general quality of life and depression were divided and evaluated; since the papers included in general quality of life and depression were assessed using the same scale, they were expressed as the mean difference. For the EuroQol-5 Dimension, which was used as the rating scale for general quality of life, being closer to 1 can be interpreted as satisfaction, 0 as a medium level, and –1 as dissatisfaction. In terms of the Patient Health Questionnaire-9, which was used as an evaluation standard for depression, 0-4 can be interpreted as normal, 5-9 as mild depression, 10-14 as moderate depression, 15-19 as severe depression, and 20 or more as severe depression.

#### General Quality of Life

**Figure 6:**
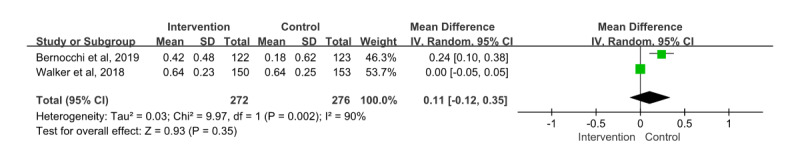
Effect size of general quality of life. IV: inverse variance [34,35].

A total of 3 papers [30,34,35] were measured using the EuroQol-5 Dimension rating scale. A paper [30] was excluded because it presented the outcome values for differences at the baseline. Therefore, the remaining 2 papers were included in the meta-analysis; a total of 548 patients participated and provided information on their general quality of life. The mean difference showed an effect size of 0.11 (95% CI –0.12 to 0.35). In addition, the effects of the intervention and control groups were not statistically significant (*z*=0.93; *P*=.35), while the heterogeneity among the papers was considerable (*χ*^2^_1_=9.97; *P=*.002; *I*²=90%; Figure 6).

#### Depression

**Figure 7:**
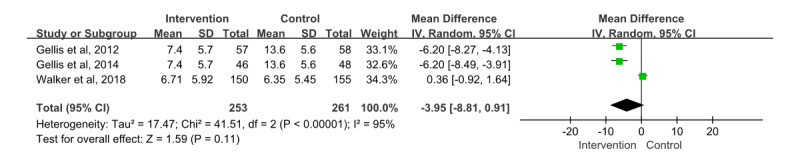
Effect size of depression. IV: inverse variance [32,33,35].

A total of 3 papers [32,33,35] were included in the meta-analysis, in which 514 patients participated and provided information on depression. For the rating scale, all 3 papers were evaluated using the Patient Health Questionnaire-9, in which the mean difference showed an effect size of –3.95 (95% CI –8.81 to 0.91). Furthermore, the effects of the intervention and control groups were not statistically significant (*z*=1.59; *P*=.11), while the heterogeneity among the papers was considerable (*χ*^2^_2_=41.51; *P*<.001; *I*²=95%; Figure 7).

### Impact of Digital Health Care Intervention on Medical Factors

In 2 papers [32,35], the effect of digital health care interventions on medical factors was assessed, and the number of hospital days was evaluated as a subgroup.

**Figure 8:**
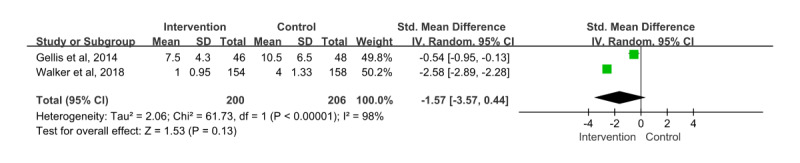
Effect size of hospital days. IV: inverse variance [32,35].

A total of 406 patients participated and provided information on the number of hospital days, and the SMD for this showed an effect size of –1.57 (95% CI –3.57 to 0.44). In addition, the effects of the intervention and control groups were not statistically significant (*z*=0.91; *P*=.36), and the heterogeneity among the papers was considerable (*χ*^2^_1_=61.73; *P*<.001; *I*²=98%; Figure 8).

### Narrative Synthesis

The 3 papers included in this study were not included in the meta-analysis because they did not present meaningful results. Regarding self-management factors, Lau et al [30] implemented home blood pressure monitoring in older patients with hypertension for 12 months. The intervention group had a higher rate of systolic blood pressure <110 mmHg, a conservative indicator of hypotension, than that of the control group, suggesting the potential effectiveness of home blood pressure monitoring [30]. Regarding medical factors, Soran et al [29] conducted a heart failure monitoring system in older patients with cardiac insufficiency for 6 months. The mean 6-month medicare cost for the intervention participants was estimated at US $17,837 and that of the control group at US $13,886; thus, the cost for the patients assigned to the intervention group was higher, suggesting no cost benefit. This may have been due to differences in the baseline demographics of the population [29]. Regarding medical factors, Tchalla et al [27] implemented a remote monitoring program for 12 months for older patients with chronic diseases. A total sample size of 536 participants was included, with the rehospitalization rate of the intervention group at 40.4% (108 people) and that of the control group at 48.7% (130 people), which showed a significant difference, suggesting the effectiveness of remote monitoring [27].

## Discussion

### Principal Findings

In this study, we aimed to confirm the properties of the intervention effect through systematic literature reviews on the effect of digital health interventions for older adults with chronic diseases living alone and to identify the effect size of intervention factors through meta-analysis. Existing studies have mainly confirmed the effects of digital health interventions targeting adults ages 18 years and older or middle-aged people aged 50-60 years [37,38]. Clear evidence of the effectiveness of digital health interventions for those aged 65 years and older has not yet been established. Accordingly, in this study, we systematically analyzed the effects of digital health interventions for older adults with chronic diseases living alone, published until October 2024, without limiting the publication year. In total, 11 papers were selected based on self-management, quality of life, and medical factors, and a meta-analysis was conducted on 8 papers. Digital health interventions were found to be effective in self-management factors for older adults with chronic diseases living alone. This suggests that digital health focuses on disease prevention and management through an increase in physical activities and the improvement of health management; thus, if effective disease management can be achieved through self-management in older adults living alone, it can be used as an alternative measure for nursing and caregiving for older adults living alone.

### Interpretation of Meta-Analysis Results on Self-Management Factor Effects

Among self-management factors, moderate-to-vigorous physical activity represented statistically significant results. This indicates that digital health interventions are effective in maintaining or improving the physical activity levels of older adults living alone. Considering that digital health is effective in improving moderate-to-vigorous physical activity in older adults living alone, the usefulness of digital health in promoting physical activity can be emphasized. These results can serve as an important basis to support the contention raised in previous studies that mHealth apps can increase the physical activities of older adults; in particular, increased physical activity among older adults with chronic diseases living alone is expected to improve their health and help prevent chronic diseases [39].

However, the results showed that the effectiveness of digital health interventions on systolic blood pressure and LDL cholesterol levels was not significant. Comprehensive differences existed in the number of populations and intervention periods between the 2 studies. Furthermore, the measurement time, environmental conditions, and variations due to the psychological effects of the patient affected the results [40-42]. Richard et al [36] suggested that among the population who participated in the study, those with a stronger desire for health were more likely to take statins and antihypertensive drugs more frequently or to participate in other cardiovascular risk reduction programs. Sun et al [28] did not consider the personal and family medical history and emphasized uncertainty in the data on diet and caloric intake.

In summary, a customized digital health system and integrated caring health care policy are needed to maximize the preventative effect on systolic blood pressure and LDL cholesterol. Personalized guidance is provided through the digital health system, taking into account an individual’s health status and habits. We can expect to prevent chronic diseases by improving personal eating habits, lifestyle habits, and blood pressure and cholesterol management. To improve the access of older adults living alone to digital health, digital health must be combined with existing medical service delivery methods, and active participation must be encouraged by reinforcing education and support for the use of digital health. Digital literacy education can particularly enhance self-management capabilities through digital health technologies. As digital literacy is essential for the success of digital health interventions, low digital literacy among older adults can be a major barrier to chronic disease management, requiring targeted education and support. Furthermore, community care, which is expanding to manage chronic diseases among older adults living alone, should integrate digital health to strengthen their self-management skills. To support this, the government must establish policies that connect community care with digital health and provide digital health education programs for older adults living alone. By supplementing the health care workforce that can provide this, the government will be able to ensure health promotion and care continuity for older adults living alone.

### Interpretation of Meta-Analysis Results on Impact of Quality of Life Factors

In studies on general quality of life and depression, digital health interventions were found to have no significant effect on older adults living alone. In terms of general quality of life, the combined results of the 2 papers did not show a significant effect on digital health. The monitoring method, characteristics of chronic diseases, and other factors may have influenced the results. In other words, the monitoring methods used in different studies and the differences depending on chronic diseases must be comprehensively considered. In addition, in terms of depression, digital health interventions did not show a significant effect on improving depression in older adults with chronic diseases living alone. This suggests that the methods or properties of digital health applied in each study may not have had a sufficient effect on depression among older adults living alone.

In a study by Bernocchi [34], a digital health system was applied that could immediately relay information to a nurse through remote monitoring, even after discharge, in the event of an emergency. This had a positive effect on improving the quality of life of older adults with chronic diseases. However, in a study conducted by Walker [35] on patients with chronic obstructive pulmonary disease, it was found that when a medical alert occurred during the digital health monitoring process, it took a considerable amount of time to establish contact [35]. Such contact delays in urgent medical situations negatively impact patient health and safety, suggesting the need for improvements in the emergency response to digital health. Furthermore, for patients with COPD, depression-related factors may occur due to worsened respiratory function, difficulties in daily life, and limitations in social participation rather than the problems of the disease itself. In this regard, the study results suggest that for older adults living alone who already have chronic diseases, there are limits to improving their quality of life according to the type of chronic disease, psychological condition, and so on.

In summary, the systematic segmentation of disease-specific monitoring systems is required to improve the quality of life factor. The main measurement factors for each disease included heart rate in patients with heart disease, respiratory rate in patients with respiratory disease, and blood sugar and food intake in patients with diabetes. Based on these key measurement factors, by implementing monitoring for each chronic disease group and then providing a variety of treatment methods, such as self-management, medication, physical therapy, and breathing exercises appropriate for the individual, patients can improve their physical and mental quality of life. Furthermore, the integrated management of chronic diseases and mental health must be implemented. Older adults with chronic diseases living alone could become patients with multiple chronic diseases. It seems that the increase in chronic diseases could be combined with social isolation and is thus expected to worsen mental health. Therefore, for effective integrated management, cooperation among various experts such as doctors, nurses, and social workers is necessary, and the establishment of a community care system is important. This is a major task in modern society, and if this integrated management system is successfully established, an improvement in patients’ mental health and chronic diseases and an improvement in the quality of medical services can be expected. In addition, it can greatly contribute to preventing patients' social isolation and providing a better quality of life.

### Interpretation of Meta-Analysis Results on the Impact of Medical Factors

Hospital days, a subgroup of medical factors, were found to have a smaller population in the digital health intervention group than in that of the control group, which did not show statistical significance. We believe that this was because the differences in the total population and intervention period in each of the 2 integrated papers had a significant impact on the results. In addition, this can be attributed to differences in the outcome measurement periods. In the study by Gellis et al [32], integrated telehealth education and activation of mood were evaluated to improve the chronic disease of cardiac insufficiency and chronic obstructive pulmonary disease and the accompanying depression in a home health care environment; the intervention period was 3 months, but the measurement of hospital days was performed 12 months after the baseline. On the other hand, the study by Walker et al [35] assessed the effectiveness of home monitoring in older patients with chronic obstructive pulmonary disease and comorbidities, in which the intervention period was 9 months. In addition, measurements of hospital days were performed immediately after the intervention period. Therefore, the difference in the time at which the number of hospital days was assessed between the 2 studies appeared to have influenced the effect size. However, the difference in the number of days in the hospital between the intervention and control groups may not be due to the effect of the digital health intervention on patient treatment but rather may be due to changes in the hospital workflow following the digital health intervention, so it needs to be interpreted cautiously. Nevertheless, in the case of older adults living alone, hospitalization may not occur due to a lack of guardians or difficulty moving around, and the intervention effect is judged to be insignificant. Thus, it is necessary to continuously verify the effectiveness of providing community care and caring services based on digital health for older adults living alone who have difficulty traveling to hospitals. Furthermore, Maximizing the synergy between telemedicine and digital health is crucial for improving patient care and reducing health care costs. Real-time monitoring allows health care providers to offer timely diagnoses and treatments, especially for older adult patients with chronic conditions, leading to better health outcomes. Continuous tracking of health indicators helps prevent complications and reduces the need for hospital visits, improving resource efficiency. By enhancing communication and data sharing between patients and medical teams, the integration of these technologies can further optimize health care delivery and lower medical expenses.

### Limitations and Strengths

The limitations of this study were as follows. First, in assessing the quality of papers, the Blinding of Outcome Assessment was performed well in most studies; however, the Blinding of Research Participants and Personnel was not implemented. Since the major outcome was measured using a subjective assessment tool, awareness of the intervention may have affected blinding. Second, only a few studies were included in this meta-analysis. A total of 8 papers were included in the overall meta-analysis, but as a result of dividing the studies into subgroups, only 2-3 articles were included and analyzed per group. There were limitations to the analysis owing to the relatively small number of articles. Third, heterogeneity was observed among the included studies. The intervention period varied between the studies, ranging from 3 to 18 months, and the timing of the outcome measurements differed slightly for each study. In particular, the number of participants in each study was very diverse, ranging from a minimum of 33 to a maximum of 2451. Therefore, the number of papers included per study group was relatively small, which may have resulted in a high degree of heterogeneity. In addition, due to the small number of papers, the test for heterogeneity was not presented separately. Fourth, we did not conduct a separate manual search, as the main literature was likely included through a highly reliable database. However, we cannot entirely rule out the possibility that some relevant studies were missed. Therefore, follow-up research incorporating a manual search is recommended.

Despite these limitations, this study confirmed the clinical effects of digital health on older adults living alone through a standardized, systematic literature review. To respond to current social problems, digital health has shown positive effects in promoting physical activities and managing chronic diseases in the self-management of older adults living alone, which will contribute to improving their health and quality of life. From this perspective, digital health can play an important role in replacing nursing and caring for older adults living alone. In the future, considering the difficulty that older adults living alone have in maintaining voluntary long-term activities, it will be necessary to continuously monitor older adults living alone through community care by applying digital health measures. Furthermore, follow-up studies are needed to explore various intervention methods to activate digital health in the health care field, which will contribute to patient-centered medical care services and a reduction in national medical costs. Ultimately, digital health interventions will move in a positive direction to manage the health of older adults living alone more effectively and improve their quality of life.

### Conclusion

This study confirmed the effectiveness of digital health interventions for older adults with chronic diseases living alone. A significant effect was found for moderate-to-vigorous physical activity among the self-management factors, but other factors did not display statistically significant results, which showed that they improved after the digital health intervention. In other words, digital health–based health care can help maintain a healthy lifestyle for older adults living alone and prevent chronic diseases. In addition, to expand the acceptance of digital health among older adults living alone, it is necessary to strengthen their digital literacy. The number of health care personnel needs to be expanded for this purpose. Therefore, a combination of digital health and local community-based care services is essential to improve the quality of life of older adults living alone and reduce their medical costs. As such, the use of digital health can serve as an inclusive technology that can supplement insufficient health care personnel and reduce social costs at the same time.

## Appendix

Multimedia Appendix 1 PRISMA (Preferred Reporting Items for Systematic Reviews and Meta-Analyses) checklist.

## Abbreviations

ABPM: ambulatory blood pressure monitoring
COPD: chronic obstructive pulmonary disease
HBPM: home blood pressure monitoring
HFMS: heart failure home care
LDL: low-density lipoprotein
mHealth: mobile health
MVPA: moderate-to-vigorous physical activity
PRISMA: Preferred Reporting Items for Systematic Reviews and Meta-Analyses
RCT: randomized controlled trial
SMD: standardized mean difference

## Data Availability

The datasets generated or analyzed during this study are available from the corresponding author on reasonable request.
